# A Case of Atrioventricular Block Potentially Associated with Right Coronary Artery Lesion and Ticagrelor Therapy Mediated by the Increasing Adenosine Plasma Concentration

**DOI:** 10.1155/2018/9385017

**Published:** 2018-04-19

**Authors:** Xiaoye Li, Ying Xue, Hongyi Wu

**Affiliations:** ^1^Department of Pharmacy, Zhongshan Hospital, Fudan University, Shanghai, China; ^2^Department of Cardiology, Zhongshan Hospital, Fudan University, Shanghai, China

## Abstract

**Purpose:**

To report a case of atrioventricular block (AVB) which might be associated with the right coronary artery lesion and the novel oral antithrombotic drug ticagrelor mediated by the increasing adenosine plasma concentration (APC).

**Case Report:**

A 65-year-old man was given loading dose of ticagrelor (180 mg) before coronary angiography with total thrombotic occlusion of right coronary artery and one stent was implanted. On second day after successful percutaneous coronary intervention, ECG monitoring showed second-degree (Mobitz type I) AVB with prolonged PR interval (299 ms). Hypothesis was drawn that elevated APC levels caused by ticagrelor would be the reason for AVB after excluding combination drugs or underlying disease. APC might be an indicator of this side effect caused by the P2Y12 receptor inhibitors. On fourth day after shifting to clopidogrel, the ECG showed normal sinus rhythm and PR interval depressed to 190 ms and APC dropped from 1.62 umol/L to 0.92 umol/L. The bradycardia and AVB did not occur in the three-month follow-up.

**Conclusion:**

It was important to take the ticagrelor induced bradycardia into account particularly with the myocardial infarction of right coronary artery, treated with atrioventricular block drugs after initiating ticagrelor. Also, we should shift ticagrelor to clopidogrel if AVB occurred.

## 1. Introduction

Ticagrelor produced faster and stronger inhibition of platelet aggregation than clopidogrel [1]. Most patients can tolerate it well, but some severe adverse drug reaction may occur including dyspnea and asymptomatic bradycardia after medication with ticagrelor [2]. In the PLATO trial, a high proportion of asymptomatic bradycardia symptoms (2.2%) occurred with ticagrelor therapy. But this transient and asymptomatic side effect did not need pacemaker [3].

Many animal and cell experiments disclosed that ticagrelor could inhibit the cellular uptake of adenosine through equilibrative nucleoside transporter 1 leading to the increase of adenosine plasma concentration (APC) [4]. These findings led to the hypothesis that this side effect of ticagrelor was mediated with adenosine which might slow down the conduction of atrioventricular nodes.

Here we reported a case about atrioventricular block (AVB) associated with ticagrelor therapy for acute coronary syndrome (ACS) patient who had a high level of APC which might be the mechanism of this adverse drug reaction. The patient was diagnosed with acute inferior wall ST-elevation myocardial infarction (STEMI).

## 2. Case Presentation

**Figure 1:**
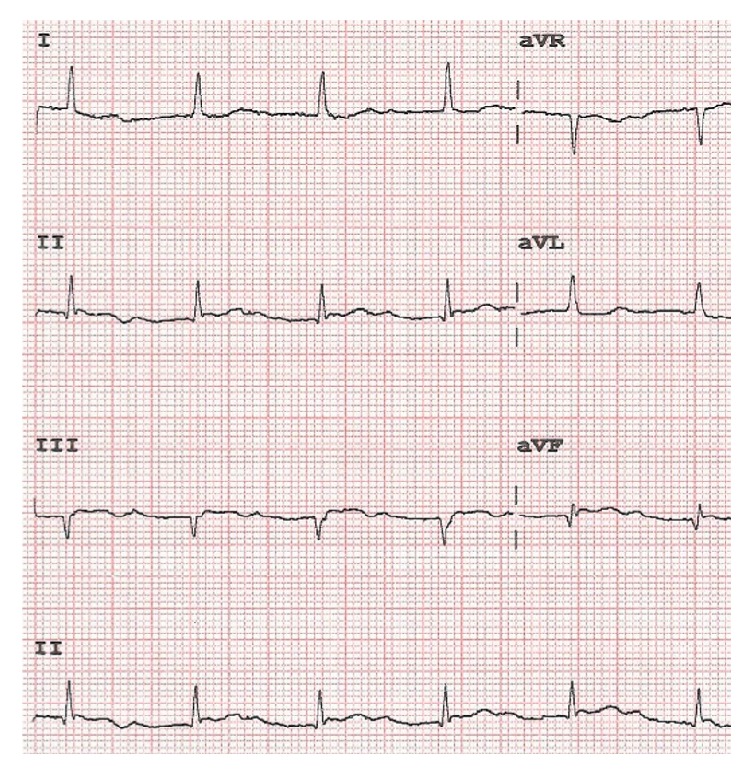
Second-degree (Mobitz type I) AVB.

**Figure 2:**
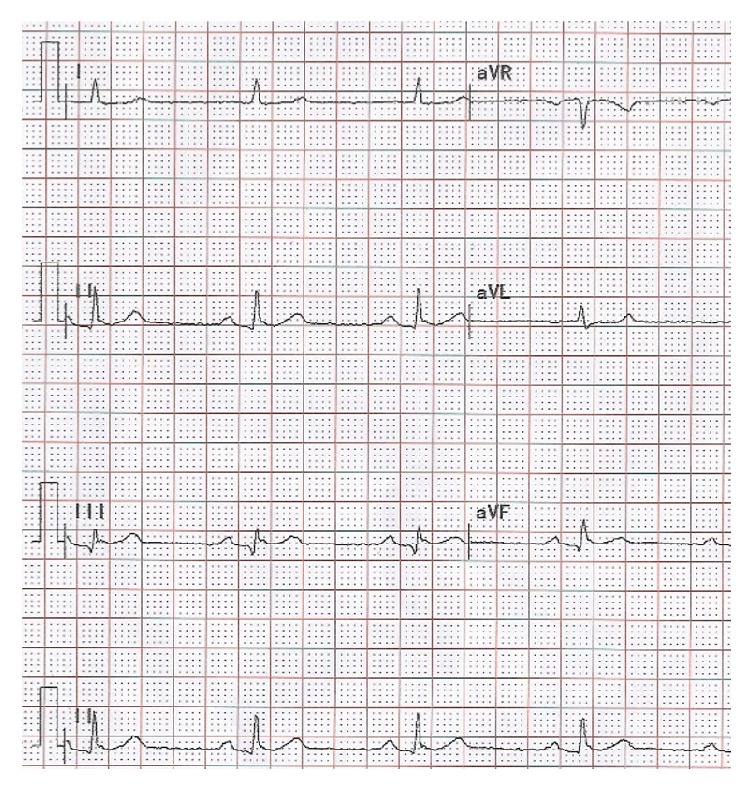
Normal sinus rhythm.

A 61-year-old male patient suffered a sudden chest pain lasting for 5 minutes accompanied with sweating, dizziness, amaurosis, nausea, and vomiting. The chest pain and tightness had no significant relief after two sublingual tablets of nitroglycerin. He had a history of hypertension of 10 years with the medication of metoprolol sustained release tablets (23.75 mg) and ramipril (2.5 mg). His admission electrocardiogram (ECG) showed normal sinus rhythm and 1 mm ST-segment elevation in lead II, lead III, and lead aVF. The repeated troponin* T* test was positive at 0.148 ng/mL and the patient was diagnosed with acute inferior wall STEMI. With initial diagnosis of STEMI, he had an emergency coronary angiography intervention after receiving a loading dose of ticagrelor (180 mg) and aspirin (300 mg). He was treated with metoprolol sustained release tablets (23.75 mg), rosuvastatin (5 mg), ramipril (2.5 mg), low molecular weight heparin (4000 IU Q12 h), and isosorbide mononitrate sustained release tablets (40 mg) for improving myocardial ischemia. The emergency coronary angiography revealed total thrombotic occlusion of right coronary artery (RCA) and one stent (2.4 mm × 18 mm sirolimus-eluting stent, EXCEL, China) was implanted in the RCA. The blood pressure (BP) was 125/72 mmHg and heart rate (HR) was 76 bpm after percutaneous coronary intervention (PCI) operation without prolonged PR interval (192 ms) and ST segment depressed to baseline. The patient was prescribed the maintenance dose of ticagrelor (90 mg bid) and the symptoms were asymptomatic and hemodynamically stable. On the second day after operation, the ECG monitoring showed second-degree (Mobitz type I) AVB with prolonged PR interval (299 ms) (Figure 1). The BP was 90/50 mmHg and HR was 45 bpm. Although beta-block might cause AVB, the patient was medicated with metoprolol sustained release tablets for many years and maintained the regular dose after onset. This side effect might be induced by ticagrelor which increased the APC. We switched the P2Y12 inhibitor from ticagrelor to clopidogrel. The APC detected by fluorescent probe adenosine assay kit (Bio Vision, Milpitas, CA 95035, USA) was 1.62 umol/L on the second day after operation. On the fourth day after shifting to clopidogrel, the ECG showed normal sinus rhythm and PR interval depressed to 190 ms (Figure 2). The BP was 104/64 mmHg and HR was 59 bpm. The APC was 0.92 umol/L. The bradycardia and AVB did not occur in the three-month follow-up.

## 3. Discussion

Ticagrelor bound directly, changed the conformation of the P2Y12 receptor, and resulted in a reversible inhibition of the receptor [5]. It can produce much faster and more efficacious P2Y12 effect than clopidogrel for the ACS patients [6]. In the PLATO trial ticagrelor was linked to increase the incidence of ventricular pauses which were predominantly asymptomatic. The incidence of acute intermittent ventricle in ticagrelor group was 6% and the incidence of intermittent ventricle was 2.2% in one-month follow-up and ticagrelor-related intermittent ventricle was self-limiting and had no impact on efficacy or safety outcomes in acute coronary syndrome (ACS) patients [3].

AVB could be caused by the delay or block conduction in any area of the atrioventricular system. AVB might be transient where the underlying etiology was reversible such as in myocarditis, in myocardial ischemia, or after cardiovascular surgery. The coronary blood flow supply of sinoatrial (SA) and AV nodes was produced by the SA and AV nodal branches most commonly originating from the RCA [7]. Therefore the reduced RCA blood flow was associated with a variety of conduction disturbances. The patient had a total thrombotic occlusion of RCA, with one stent implanted. So the RCA lesion might be responsible for this conduction disturbance in our case.

AVB could occur during the drug therapy including *β*-blockers and digoxin, or non-DHP CCBs might cause AV block, primarily in the AV nodal area. In our case, the patient was medicated with metoprolol sustained release tablets for over 10 years. So it was not logical to think this newly occurring conduction disturbance was associated with metoprolol.

Another important point related to this conduction disturbance might be the medication of ticagrelor. After medication with the loading dose of ticagrelor (180 mg), the BP was 90/50 mmHg and HR was 45 bpm. Pharmacology screening showed the inhibition of adenosine uptake into human erythrocytes as one of the most potent off-target activities of ticagrelor [4]. However this mechanism of ticagrelor was shown to have specific side effects such as bradycardia and dyspnea, which could also be triggered by APC increase. The APC that was detected by fluorescent probe adenosine assay kit decreased significantly after switching the P2Y12 inhibitor from ticagrelor to clopidogrel. The latest research discovered that the ticagrelor could inhibit the adenosine uptake by red blood cells in ACS patients, leading to the increase in APC similar to what was observed for dipyridamole [4].

Adenosine exerted cardiac electrophysiological effects through activation of A_1_R. It had a negative chronotropic effect through suppression of the automaticity of cardiac pacemakers and a negative dromotropic effect on the inhibition of AVB [8]. Adenosine was rapidly taken up by cells through sodium-independent equilibrative nucleoside transporters and sodium-dependent concentrative nucleoside transporters compared with placebo in in vitro experiment [9]. The effect of ticagrelor influencing adenosine concentrations may convey unique properties of the drug not shared by other P2Y12 antagonists such as clopidogrel, prasugrel, and cangrelor.

## 4. Conclusion

In our case, the right coronary artery lesion and medication with ticagrelor might be the main reasons for the AVB. Extreme caution and close ECG monitoring after initiation of the antiplatelet drug ticagrelor were needed in terms of development of the myocardial infarction with right coronary artery.
